# A novel automatic quantification method for high-content screening analysis of DNA double strand-break response

**DOI:** 10.1038/s41598-017-10063-0

**Published:** 2017-08-29

**Authors:** Jingwen Feng, Jie Lin, Pengquan Zhang, Songnan Yang, Yu Sa, Yuanming Feng

**Affiliations:** 1 Department of Biomedical Engineering, Tianjin University, Tianjin, 300072 China; 2 Tianjin Optical Electrical Group Ltd, Tianjin, 300211 China; 3 Tianjin Medical University Cancer Institute and Hospital, Tianjin, 300060 China

## Abstract

High-content screening is commonly used in studies of the DNA damage response. The double-strand break (DSB) is one of the most harmful types of DNA damage lesions. The conventional method used to quantify DSBs is γH2AX foci counting, which requires manual adjustment and preset parameters and is usually regarded as imprecise, time-consuming, poorly reproducible, and inaccurate. Therefore, a robust automatic alternative method is highly desired. In this manuscript, we present a new method for quantifying DSBs which involves automatic image cropping, automatic foci-segmentation and fluorescent intensity measurement. Furthermore, an additional function was added for standardizing the measurement of DSB response inhibition based on co-localization analysis. We tested the method with a well-known inhibitor of DSB response. The new method requires only one preset parameter, which effectively minimizes operator-dependent variations. Compared with conventional methods, the new method detected a higher percentage difference of foci formation between different cells, which can improve measurement accuracy. The effects of the inhibitor on DSB response were successfully quantified with the new method (p = 0.000). The advantages of this method in terms of reliability, automation and simplicity show its potential in quantitative fluorescence imaging studies and high-content screening for compounds and factors involved in DSB response.

## Introduction

DNA double-strand breaks (DSBs) are among the most harmful types of lesions in eukaryotic DNA damage and can lead to cell death after a failed repair. In that situation, the DSB response plays a crucial role for cell survival. Radiation and many chemical agents can lead to DSBs. Inhibitors of the DNA DSB response in combination with radiotherapy or chemotherapy have been used to improve the efficacy of cancer therapy. A high-content screening system with fluorescence cell image analysis has been verified as suitable for the screening of inhibitors because it enables the direct visual observation of the sub-cellular localization of factors and the interactions between them. Phosphorylation modification of histone H2AX on serine 139 (γH2AX) is an early step in cellular response to DSBs^1–3^. Foci of γH2AX are generally regarded as sensitive molecular markers of DSBs^4^. Some proteins that participate in the DNA damage response (DDR), such as 53BP1 and RAD51, are recruited to DNA damage sites in a stepwise manner and can be visualized as discrete foci after fluorescent staining^5, 6^. These foci co-localize with γH2AX, which can be used to quantify the activation of different DSB response pathways.

Foci counting is commonly used in clinical and basic research to estimate the DSB response^7, 8^. Although this method is relatively simple, it still requires human intervention for foci definition, which is potentially time-consuming and often leads to poorly reproducible outcomes. The detection sensitivity of this method is dependent on the number of DSBs per cell. High-dose radiation induces severe damage to DNA, the images of which are characterized by dense and overlapping foci and often lead to unreliable results. Furthermore, segmentation with a single threshold is not a reliable method for multi-cell images, as the fluorescence intensity varies between cells in the same field of view. Although some research has improved the accuracy of this method, it is still limited by the radiation dose^9, 10^. Given these disadvantages, the foci counting method is less than optimal for automatic analysis of the DSB response in high-content screening; thus, a universal standard high-content screening method for quantifying the DSB response is lacking. Studies have showed that foci counting of specific DSB response proteins after immunostaining is easy to implement, but these studies did not consider the differing extent of DNA damage in each cell^11^. Other reports have shown that the effects of inhibitors of signaling events in the DDR can be quantified by analyzing γH2AX foci, but the specific protein participating in this process cannot be visualized^12^. Therefore, a comprehensive quantitative method must be established to obtain more valuable information for high-content screening experimental studies.

In this manuscript, we present a simple, accurate and robust method for the comprehensive quantitative analysis of the DSB response as a practical alternative. This method was tested by estimating the effects of MS-275 (a class I/III histone deacetylase inhibitor, HDACi) in a radiation-induced DSB response and compared with other methods. MS-275 has excellent *in vivo* antitumor activity against human tumors^13^. MS-275 can not only manipulate the cell cycle by regulating the expression of some proteins but also inhibits the DDR by maintaining the acetylation level of particular acetylation sites in histones, resulting in the failure of homology-directed repair (HDR) and DSB non-homologous end joining (NHEJ)^11, 14, 15^. The DSB repair protein 53BP1 is mainly involved in the non-homologous end joining signaling pathway^16^. To realize automatic detection, image cropping was performed to obtain a sub-image of each nucleus, and the threshold for foci-segmentation for each sub-image was obtained automatically. Pixel intensity was considered to improve the accuracy and automation of this new method. Co-localization analysis was used for the first time to evaluate the effects of the inhibitor on specific DSB response proteins. Compared with other quantitative methods, the new method can better quantify dense and/or overlapping foci as long as the fluorescence signal is unsaturated. Co-localization analysis can reduce human interventions and provide a global understanding of the DSB response. Our results show that the new method can be used for quantifying the DSB response in high-content screening image analysis.

## Materials and Methods

In this study, all experiments were performed in accordance with relevant protocols and repeated three times.

### Cell culture

The human lung adenocarcinoma cell line A549 and human breast cancer cell line MCF-7 were, respectively, cultured in RPMI 1640 and Dulbecco’s modified eagle medium (DMEM) supplemented with 10% fetal bovine serum (FBS).

### Western Blotting

MS-275 was dissolved in dimethylsulfoxide (DMSO), prepared as solutions with a concentration of 20 μM and stored at 4 °C. A549 and MCF-7 cells were treated with MS-275 (in DMSO) at different concentrations for 24 hours. Total protein was isolated from cells using sodium dodecyl sulfate (SDS) buffer supplemented with protease inhibitors. The concentration of samples was measured using a NanoDrop2000 instrument (Thermo Fisher Scientific, Waltham, MA, USA). Equal amounts of samples were separated on 8–15% SDS-PAGE gels and transferred to polyvinylidene fluoride membranes. After blocking with 5% skim milk, the membranes were immunoblotted with primary antibodies overnight at 4 °C and washed with 1 × Tris buffered saline with Tween (TBST) 3 times. The secondary antibodies were added for 1 hour at room temperature. After washing with 1 × TBST, the targeted proteins were detected via chemiluminescence using ECL-plus detection (PerkinElmer Life Science, Waltham, MA, USA).

### Antibodies

The antibodies used were 53BP1 (sc-22760, Santa Cruz Biotechnology, CA, USA), H4ac (05–1355, Millipore, Temecula, CA, USA), H3K56ac (ab76307, Abcam, Cambridge, UK), β-actin (sc-1616, Santa Cruz), and γH2AX (05-636-1; Millipore). Alexa Fluor® 546 (excitation/emission: 556 nm/570 nm) and Alexa Fluor® A488 (excitation/emission: 488 nm/519 nm) (A11010, Invitrogen Life Sciences, Carlsbad, CA, USA) were also used.

### Immunofluorescent staining and image acquisition

Cells were grown in 96-well plates and separated into experimental and control groups. MS-275 (in DMSO) was added into the experimental group to a final concentration of 5 μM and equal volume of DMSO was added into the control group 24 hours before irradiation. To obtain cell images with dense and overlapping DSB foci, cells were exposed to a single dose of 10-Gy delivered using a 6-MV photon beam from a Clinac 600 CD linear accelerator (Varian Medical Systems, Palo Alto, CA, USA). The pre-experiment results (Supplementary Fig. S1) showed that the inhibitory effect of MS-275 on 53BP1 foci formation can be detected effectively at the time point of 60 min after irradiation, which is in consistence with the previous report^11^. Therefore, cells were cultured for 60 min at 37 °C with 5% CO_2_ after irradiation and fixed with 4% paraformaldehyde for 30 min at room temperature. After being washed with PBS, cells were permeabilized using 0.1% Triton-X 100 for 15 min at room temperature followed by incubating with 5% bovine serum albumin (in 1 × PBS, pH 7.4) for 1 hour for blocking. The primary antibodies were added at an appropriate dilution and incubated overnight at 4 °C. The next day, cells were washed in PBS three times before being incubated with fluorescent-labeled secondary antibody for 1 hour at room temperature. The antibodies were aspirated, and cells were washed in PBS three times. DAPI was used to label the cell nucleus.

Images were acquired using the automated fluorescence microscope platform of the In Cell Analyzer 2200 using a 40 × objective lens. Images from 6 fields per well were collected to obtain data for up to 300 cells. The pixel size was 6.5 μm × 6.5 μm. The pixel size for imaging was 2048 × 2048. The blue channel is a DAPI-stained nucleus (excitation/emission: 358 nm/461 nm). Filters selected were DAPI for nucleus, FITC for Alexa Fluor® A488 and Cy3 for Alexa Fluor® 546. The green channel and red channel represent the DSB foci (γH2AX foci) and DDR foci (53BP1 foci), respectively.

### Image cropping

DAPI counterstaining was used to show the region of nucleus. Three steps were followed in image pre-processing: 1. use Otsu thresholding^17^ to segment nuclear images and remove the elements in the image that do not represent intact nuclei with morphology operations; 2. identify the outline of every nucleus using the Canny edge detection algorithm;^18^ 3. apply the nuclear outlines to the red channel and green channel to correlate foci with specific nuclei. As the fluorescence intensity of each nucleus differs even in the same field of view, it is difficult to choose absolute threshold values for image processing. To solve this problem, the image was cropped according to nuclei using the following steps: 1. use the bounding rectangle of a nucleus as the image ROI (region of interest); 2. copy the image ROI to obtain two identical sub-images for each nucleus; 3. fill in the nucleus region in one sub-image with zero, and subtract the filled sub-image from the other sub-image to obtain one sub-image with a single cell nucleus. Foci images of a single cell nucleus from the green and red channels were obtained with the same method.

### Foci detection

Information for the foci, including the numbers and size, was extracted with fuzzy c-means clustering from each nuclear image^19^.

### Quantitative analysis

#### Automatic foci intensity quantification

An automatic quantification algorithm was developed for foci intensity measurement after detection. The summation of the foci intensity in one nucleus, represented by *I*_*sum*_, is calculated using equation (1),

1 $${I}_{sum}={\int \int }_{({\rm{x}},{\rm{y}})\in {\rm{S}}}f({\rm{x}},{\rm{y}}){\rm{dxdy}}$$

where S is the region of integration, (x, y) is the pixel coordinates, and *f*(x, y) is the intensity of pixel (x, y) distributed in the S region. The discrete form of this equation used in the digital image processing is shown in equation (2),

2 $${I}_{sum}=\sum _{({\rm{i}},{\rm{j}})\in {\rm{S}}}I({\rm{i}},{\rm{j}})$$

where (i, j) indicates the pixel in the image and *I*(i, j) represents its intensity.

#### Quantification of the co-localization of γH2AX and 53BP1

After image processing, the DSB foci image (γH2AX foci) was obtained from the green channel and the DDR foci image (53BP1 foci) from the red channel. Two objects are co-localized in one position when the pixel intensities of this position are greater than zero in both the green and red channels. We used a ratio, Ra, to represent the effects of the inhibitor on the radiation-induced DSB response capacity. Ra is given by equation (3),

3 $${Ra}=\frac{{\iint }_{({\rm{x}},{\rm{y}})\in {{S}}_{{G}}}{{f}}_{{R}}({\rm{x}},{\rm{y}})\mathrm{dxdy}}{{\iint }_{({\rm{x}},{\rm{y}})\in {\rm{S}}}{{f}}_{{G}}({\rm{x}},{\rm{y}})\mathrm{dxdy}}$$

where *S* is the nuclear region, *S*_*G*_ is the region of pixel intensity greater than zero in the green channel *S*_*G*_ = {S|*f*_*G*_(x, y)}, (x, y) is the pixel coordinates, *f*_*G*_ (x, y) is the pixel intensity of (x, y) in the red channel, and *f*_*G*_ (x, y) is the intensity of pixel (x, y) in the green channel. The discrete form of equation (3) is expressed as

4 $$Ra=\frac{\sum _{({\rm{i}},{\rm{j}})\in {S}_{G}}{I}_{R}({\rm{i}},{\rm{j}})}{\sum _{({\rm{i}},{\rm{j}})\in S}{I}_{G}({\rm{i}},{\rm{j}})}$$

where (i, j) is the subscript of the pixel in the image, *I*_*R*_ is the intensity of this pixel in the red channel, *I*_*G*_ is the intensity of this pixel in the green channel, and *S*_*G*_ is the region of pixel intensity greater than zero in the green channel *S*_*G*_ = {S|*I*_*G*_(*i, j*) > 0}. With this method, only the pixel intensity of 53BP1 foci co-localized with γH2AX was calculated.

### Statistical analysis

Independent samples’ Wilcoxon rank-sum tests were used for significant difference analysis, as cells were randomly divided into two groups and treated with different experimental conditions. A minimum sample size of 255 cells was used. A p value less than 0.05 was considered to be significant. Error bars are defined as the mean ± standard deviation (SD). The percentage difference was calculated to estimate the sensitivity of different methods for foci quantitation. The coefficient of variation (CV) was calculated to analyze data variation between different methods for DSB response measurement.

### Availability of materials and data

No datasets were generated or analyzed during the current study.

## Results

### Determination of MS-275 concentration

**Figure 1:**
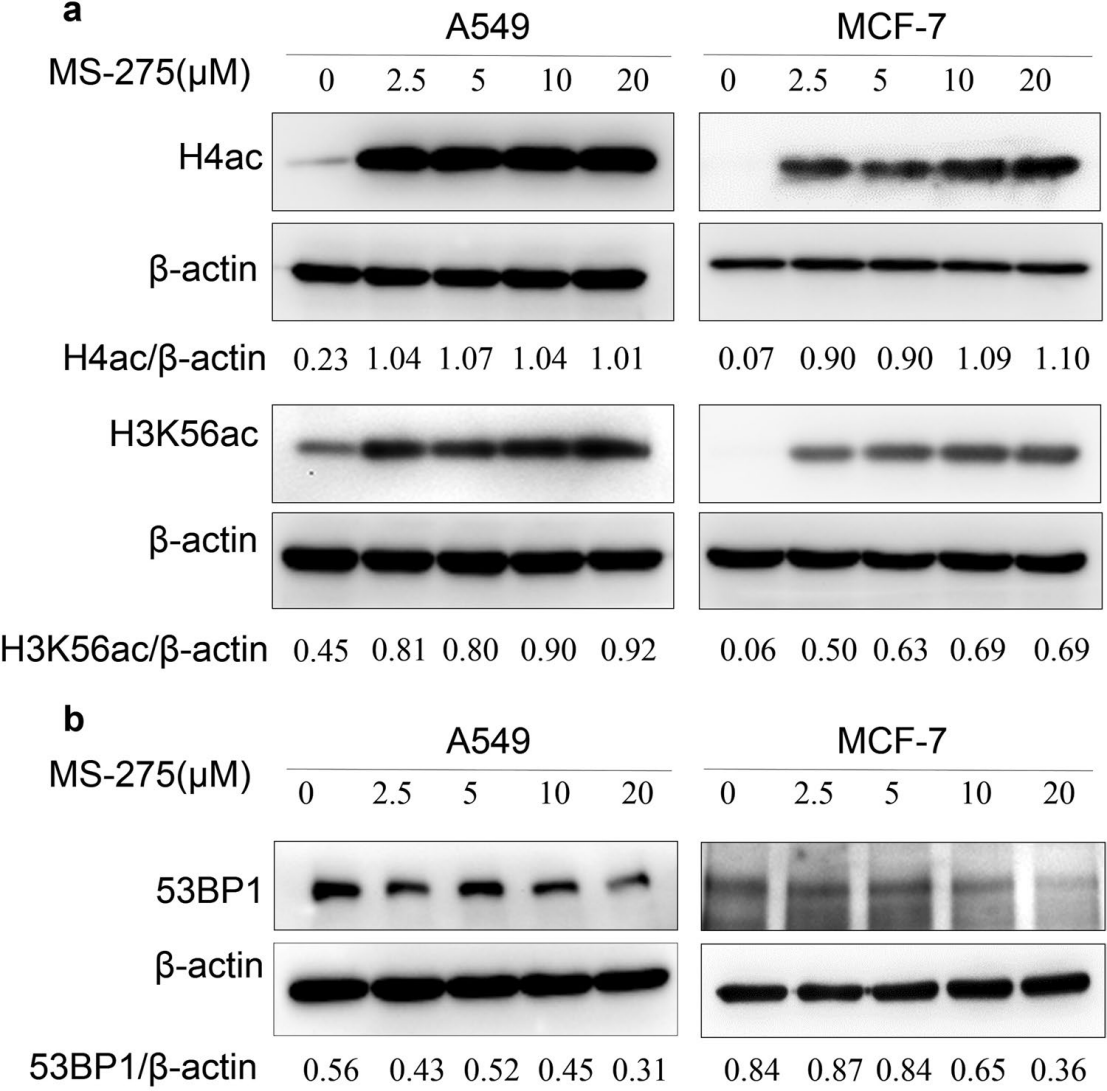
Induction of H4ac and H3K56ac by MS-275 and expression level of 53BP1. **(a)** A549 and MCF-7 cells were incubated with different doses of MS-275 (in DMSO). H4ac and H3K56ac were substantially increased and showed a positive correlation with drug concentration. (**b)** The expression level of 53BP1 was not affected by MS-275. (Fig. 1a and b are cropped gels/blots, the full-length gels/blots are included in the Supplementary Information).

Western blot assay was used to determine the appropriate experimental dose of MS-275. The results showed that H4ac and HK56ac substantially increased after treatment with MS-275 (in DMSO) (Fig. 1a), and the expression level of 53BP1 was not obviously changed (Fig. 1b). Based on the results of the quantitative analysis, we found that the effects of 2.5 µM and 5 µM MS-275 on histone deacetylation and 53BP1 expression were similar. Generally, low-dose of agents leads to less cell cytotoxicity in biological or clinical experiments. However, the agent concentration is not a sensitive factor in this study, and appropriate concentration of the agent is beneficial to the stability and reproducibility of the experiments. To reduce the sampling error and obtain stable and effective results, a 5 μM dose was chosen in our subsequent experiments.

### Automatic foci segmentation

**Figure 2:**
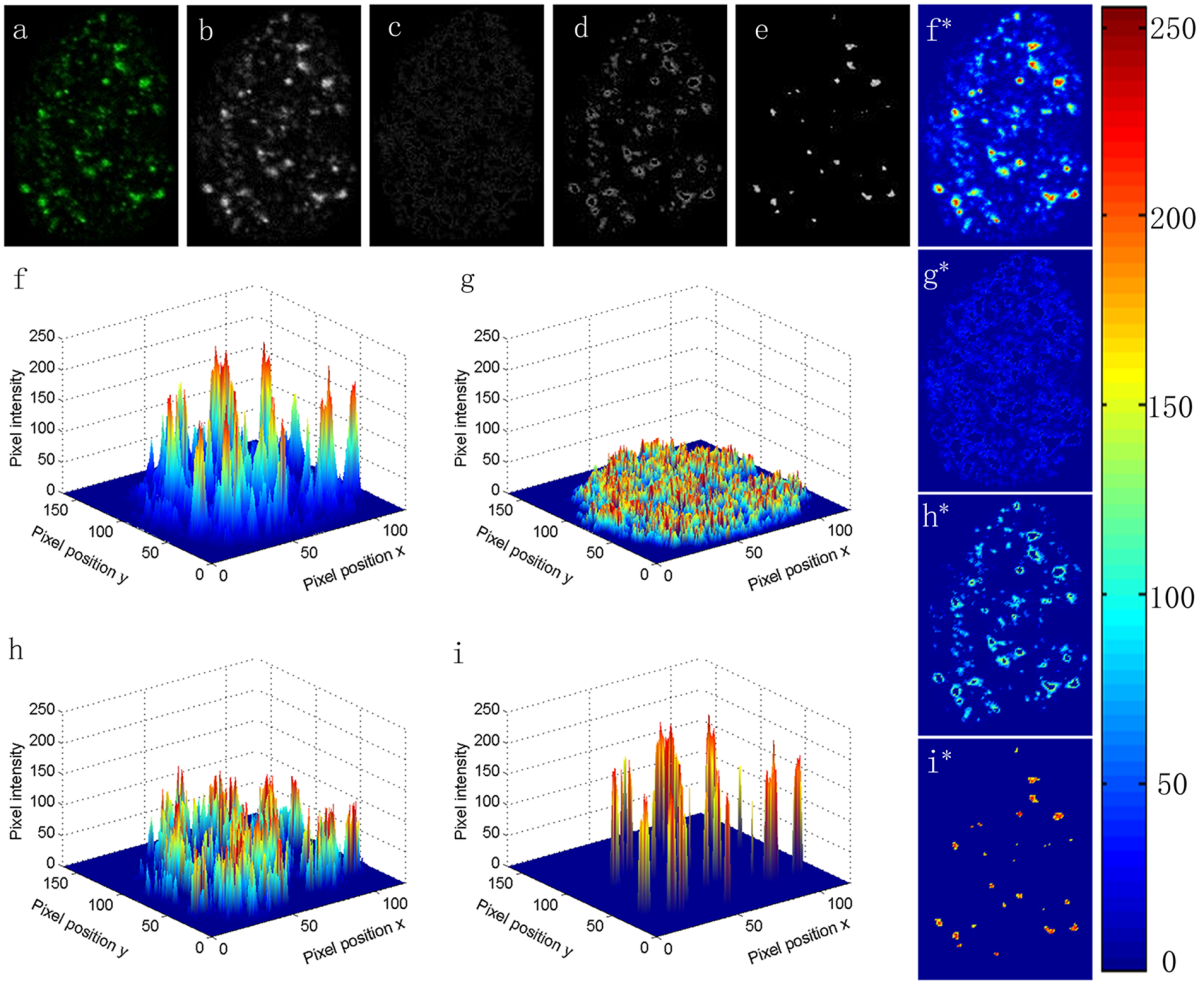
Automatic foci segmentation with fuzzy c-means clustering. **(a)** γH2AX foci image of a MCF-7 cell nucleus randomly chosen from the control group. (**b)** Gray level image of (**a**). (**c)** Gray level image of the first cluster after processing with fuzzy c-means clustering. (**d)** Gray level image of the second cluster after processing with fuzzy c-means clustering. (**e)** Gray level image of the third cluster after processing with fuzzy c-means clustering. (**f)** Three-dimensional display of (**b)**. (**g)** Three-dimensional display of (**c)**. (**h)** Three-dimensional display of (**d)**. (**i)** Three-dimensional display of (**e)**. (f*–i*) Two-dimensional pseudo-images of (**f**–**i)**.

Figure 2 shows the processing procedure and effect of fuzzy c-means clustering. Figure 2a is an original pseudo-color image of γH2AX foci in one cell nucleus. The pseudo-color image was converted to gray level image as shown in Fig. 2b. Fuzzy c-means clustering was applied to segment the gray level image into three clusters according to the grayscale levels to extract the foci regions (Fig. 2c,d,e). Three-dimensional and two-dimensional pseudo-color images of the original image and the clustering results are displayed in Fig. 2 as well (Fig. 2f,g,h,i,f*,g*,h*,i*). Compared with the other clusters, pixel intensities of the images in the third cluster were apparently much higher, which were successfully extracted and taken as foci information in the quantitative analysis (Fig. 2e,i,i*).

### Automatic foci quantitation

**Figure 3:**
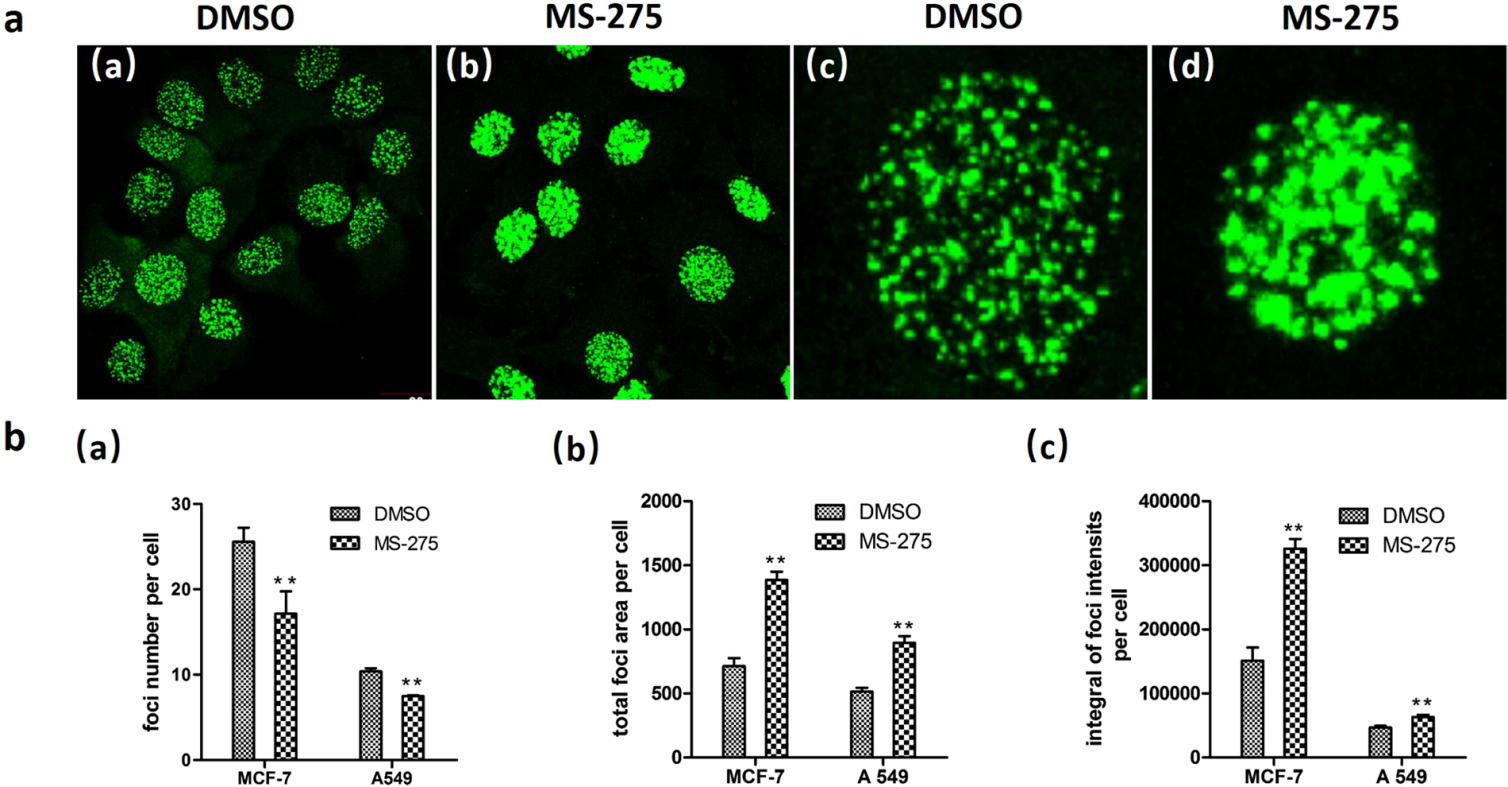
Results of automatic γH2AX foci quantification for images of MCF-7 and A549 cells from high-content screening. Cells incubated with DMSO and/or MS-275 (in DMSO) were exposed to 10-Gy radiation and then were cultured for another 1 hour before immunostained with antibodies against γH2AX and 53BP1. Quantification was performed using three different quantitative methods. (**a)** (a) Whole visual field of cells treated with DMSO (MCF-7). (b) Whole visual field of cells treated with MS-275 (in DMSO). (c) Single cell nucleus image from cells treated with DMSO. (d) Single cell nucleus image from cells treated with MS-275 (in DMSO). (**b)** (a) Quantification of γH2AX foci numbers. (b) Quantification of γH2AX foci areas. (c) Quantification of γH2AX foci intensity. Error bars represent standard deviation (SD) of three independent experiments, each based on more than 100 cells under each treatment condition. Significant differences were observed compared to DMSO-treated cells, and the p values of all quantitation methods were the same (p = 0.000). The data were derived from three independent experiments. **p < 0.01.

After foci-segmentation, we quantitatively analyzed the number, total area and integral of pixel intensities of γH2AX foci in each sub-image to determine whether the pixel intensity could be used for foci quantitation. Figure 3a shows that cells treated with MS-275 leads to severer DSBs as compared with cells treated with DMOS only after being exposed to 10-Gy radiation, which is in agreement with previous reports^11, 20^. Quantitative analysis results showed that the foci number of γH2AX per cell was significantly decreased (Fig. 3b(a)). However the total area and the integral of pixel intensities of γH2AX foci per cell in the experimental group were significantly higher than those in the control group in both A549 (p = 0.000) and MCF-7 (p = 0.000) cell lines (Fig. 3b(b)(c)). This result indicated that the last two methods reliably detected increased induction of γH2AX foci formation by MS-275 in combination with radiation. A possible explanation for this finding is that most images of the cells treated with MS-275 in combination with 10-Gy radiation are characterized by overlapping and conglutinating foci, which interfered with the accuracy of the foci counting method.

### Comparison of different methods on foci quantification

To investigate the sensitivity of the integral of foci pixel intensities for identifying the DSB response, cells from experimental and control groups were randomly chosen, and quantitative results were obtained with the three methods and compared.

**Figure 4:**
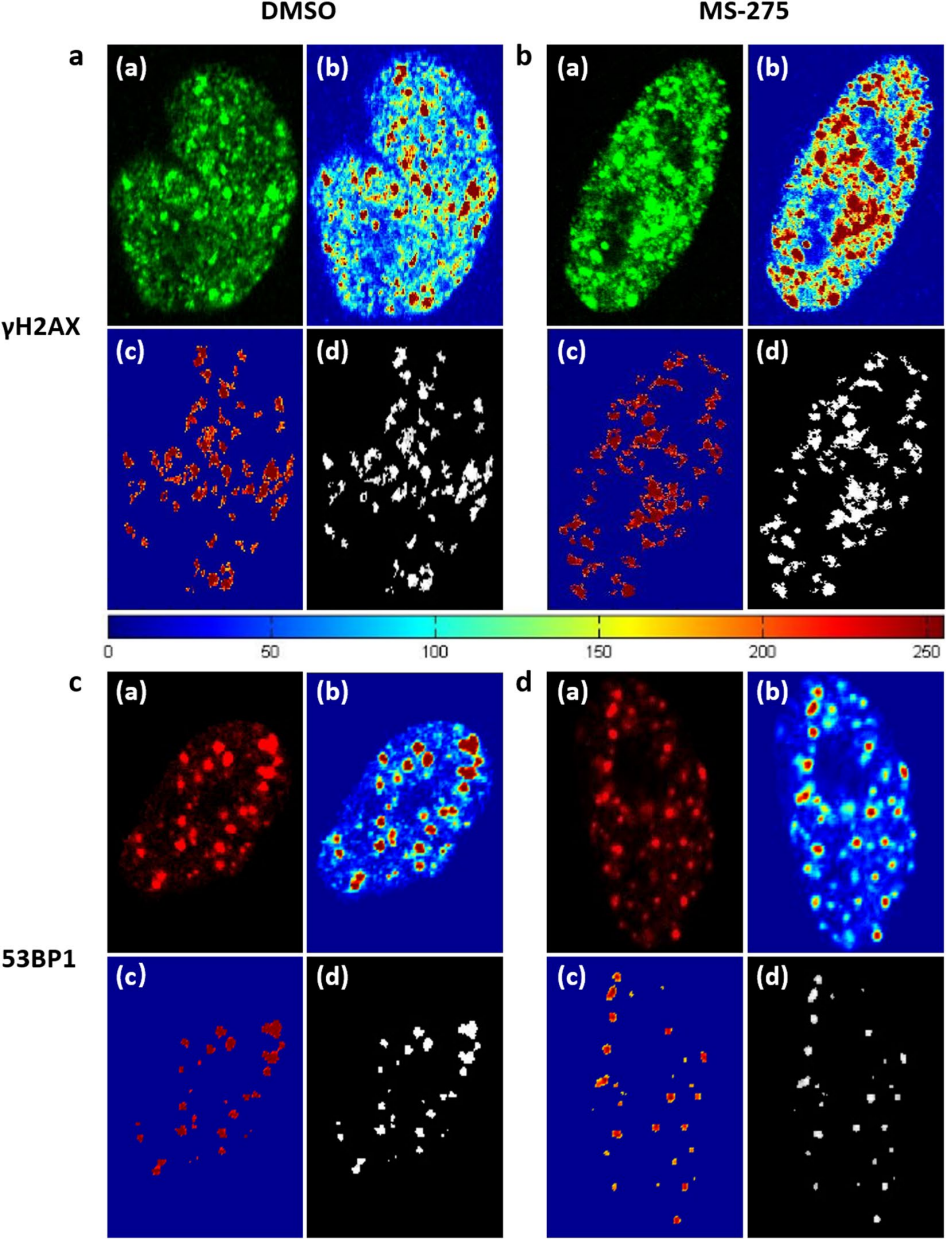
Quantification of foci formation. (**a)** Cells from the experimental group labeled with γH2AX antibody. (**b)** Cells from the control group labeled with γH2AX antibody. (**c)** Cells from the experimental group labeled with 53BP1 antibody. (**d)** Cells from the control group labeled with 53BP1 antibody. (a) Fluorescence image of a cell nucleus. (b) Pseudo-color image of (a) based on the grayscale level. (c) Pseudo-color image of (d). (d) Extracted foci image.

The results showed that both the foci numbers and total foci areas of γH2AX and 53BP1 between the cells from the experimental (MS-275 in DMSO) and control (DMSO) groups were similar (Fig. 4a(d),b(d),c(d),d(d)). However, the pixel intensities of the foci were markedly different. As shown in Fig. 4, the pixel intensity of the γH2AX foci of the cell from the experimental group was much higher than from the control group, and the trend in pixel intensity variation of 53BP1 foci was opposite, in agreement with previous reports^11^. MS-275 can increase DNA DSBs and inhibit the DSB response^11, 20^ (Fig. 4a(b),b(b),c(b),d(b)). Detailed statistical analysis is shown in Tables 1 and 2. Percentage difference is given by equation (5)

5 $$diff=abs({\rm{data}}\,1-{\rm{data}}\,2)/\,{\rm{\min }}({\rm{data}}\,{\rm{1}},{\rm{data}}\,{\rm{2}})\ast 100 \% $$

where *diff* is the percentage difference between the results obtained using different methods, *data1* is the result of the treated cell (Fig. 4a,c), *data2* is the result of the control cell (Fig. 4b,d) and *min (data1, data2)* is the minimum value between *data1* and *data2*. The results from different methods were normalized, and the percentage differences were compared to estimate the ability of different methods in identification of the fluorescence foci in cells under different treatment conditions. These results showed that the foci counting method can yield false negatives when the DSB foci were dense and overlapping. The statistical analysis showed that the integral of foci pixel intensities demonstrated better recognition than total foci area. This result indicates that the integral of foci pixel intensities is a useful index for quantifying foci formation in DNA DSBs, and the detection accuracy is only slightly affected by the density of the foci (Tables 1 and 2).

**Table 1:** Comparison of different quantitative methods on for foci quantification in DNA damage.

	Foci number	Total foci area	Integral of foci intensities
Nucleus a	46	2054	522845
Nucleus b	43	2754	920712
Percentage difference (%)	6.97	55.8	76.09

Nucleus a corresponds to Fig. 4a.

Nucleus b corresponds to Fig. 4b.

**Table 2:** Comparison of different quantitative methods on for foci quantification in 53BP1 foci formation.

	Foci number	Total foci area	Integral of foci intensities
Nucleus c	27	637	154479
Nucleus d	30	462	92493
Percentage difference (%)	11.11	37.88	67.02

Nucleus c corresponds to Fig. 4c.

Nucleus d corresponds to Fig. 4d.

### Quantitative analysis of MS-275 effects on 53BP1 foci formation at DSBs

**Figure 5:**
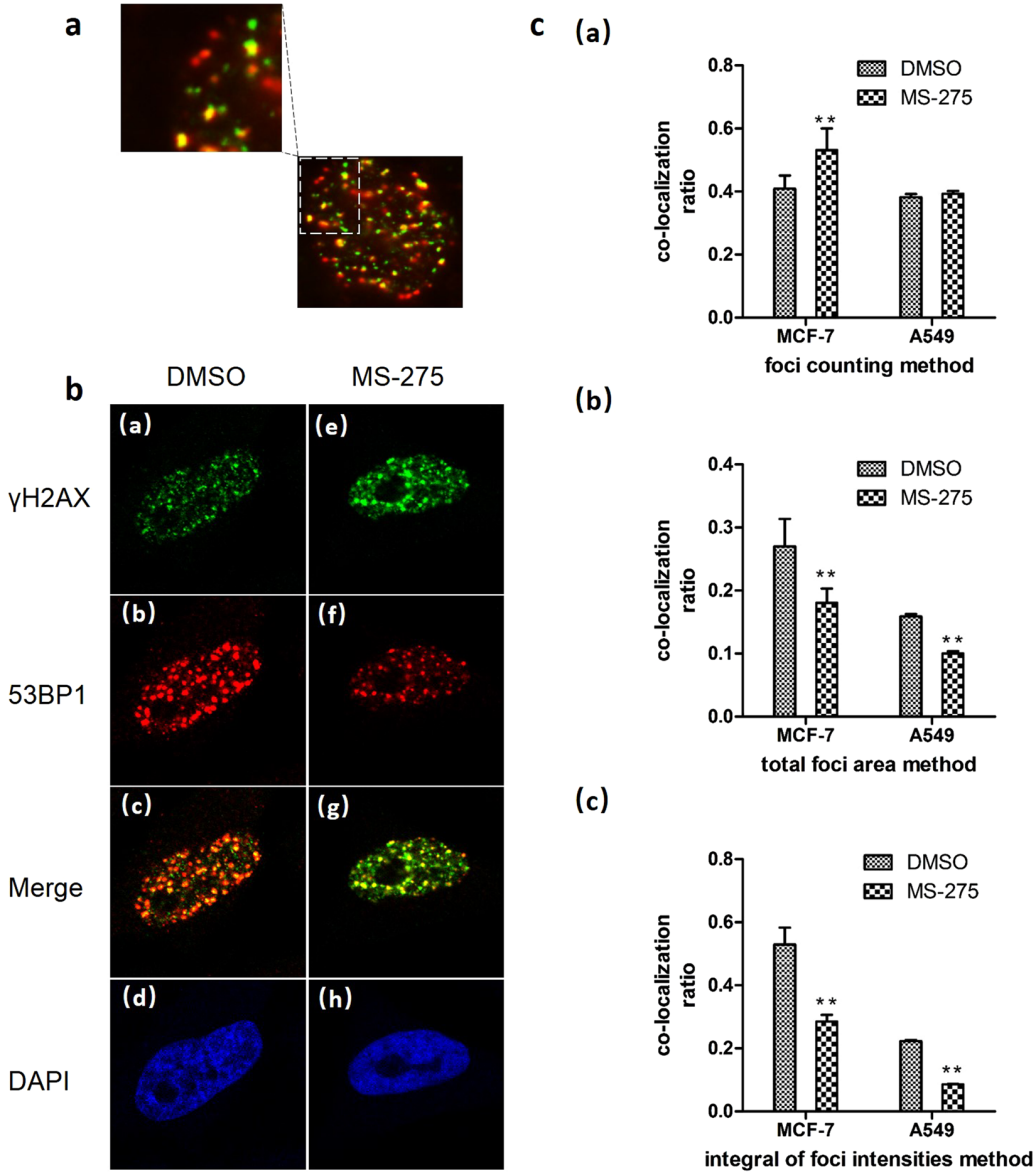
Effects of MS-275 on 53BP1 foci formation at DSBs. Cells were incubated with DMSO and/or MS-275 (in DMSO) for at least 24 hours before exposure to 10-Gy radiation and then were cultured for another 1 hour before fixed and immunostained with antibodies against 53BP1 and γH2AX. (**a)** Co-localization of 53BP1 and γH2AX. Image acquired using an Olympus IX71 microscope. (**b)** Co-localization of 53BP1 and γH2AX. Image acquired using an In Cell Analyzer 2200. (**c)** Quantitative analysis of effects of MS-275 on 53BP1 foci formation at DSB sites. (a) Quantitative analysis using the foci counting method. (b) Quantitative analysis using the total foci area method. (c) Quantitative analysis using the integral of foci intensities method, i.e., new method. Error bars represent SD of three independent experiments, each based on more than 100 cells under each treatment condition. A significant difference was observed compared to DMSO-treated cells. The data were derived from three independent experiments. **p < 0.01.

In this study, a new method based on the extensive co-localization of DSBs and DDR proteins was established to standardize the quantification of the DNA DSB response. Only the DDR protein foci that co-localized with γH2AX were calculated. The ratio of the DDR protein to γH2AX was defined as the co-localization ratio. This arithmetic operator can effectively avoid the influence of the foci in different focus planes (Fig. 5a). The new method was used to estimate the effect of MS-275 on inhibiting 53BP1 recruitment to DSBs. The foci number, total foci area and integral of foci intensities per cell were calculated and compared. As shown in Fig. 5, 1 hour after radiation at 10-Gy, 53BP1 foci formation was significantly inhibited by MS-257 in both MCF-7 (p = 0.000) and A549 (p = 0.000) cells, which is in agreement with previous reports^11^ (Fig. 5b,c(b) and (c)). Figure 5c(a) shows that 53BP1 foci formation increased after treatment with MS-275 (in DMSO) in both MCF-7 (p = 0.000) and A549 (p = 0.529) cell line, completely opposite to previous reports. As discussed in the previous section, the foci counting method exhibits limitations in being able to analyze images with dense and overlapping foci. The coefficient of variation of the new method is 4.14%, which is much lower than that of the foci area method (CV% = 8.97%) and indicates that the integral of foci intensities is a more stable index than total foci area in estimating the accumulation of DNA DSB repair proteins.

## Discussion

According to reports, high-content screening systems have been widely used in investigations estimating the potential of compounds as sensitizers in radiation and chemotherapy^12, 21, 22^. However, there is a lack of an automatic, accurate and comprehensive quantitative method for image analysis, which is slightly affected by the degree of DNA damage.

The method presented in this manuscript employs a unique combination of techniques for object detection in images with cells that have different fluorescence intensity, and the method shows several advantages. The application of image cropping and fuzzy c-means clustering—which considers variation in background signal between different cells under the same image acquisition conditions—realizes accurate segmentation. The number of clusters is the only parameter that must be manually preset based on visual judgment. This method enables easy implementation in high-content screening studies. Integrated foci pixel intensities were used as a key index to avoid overestimating and underestimating the foci and to improve recognition in analyzing images with dense and overlapping foci. The concept of a co-localization ratio was introduced to precisely quantify the DNA DSB response for the first time. These advantages enable unbiased evaluation of the DSB response.

The new method was verified and compared to currently used techniques using a well-known DNA damage repair inhibitor, histone deacetylase inhibitor MS-275, in combination with radiation. Quantitative analysis shows that MS-275 increases radiation-induced DNA damage in A549 and MCF-7 cell lines. At a concentration of 5 μM, MS-275 can effectively inhibit 53BP1 foci formation on DSBs but does not affect the expression of 53BP1 protein. These results are in agreement with previously reported results and indicate that the new method can effectively detect and quantify the DSB response in the experimental and control groups and recognize the variation between them. Statistical analysis shows that the new method has advantages of high accuracy, high recognition and stability compared with other methods in dealing with images of cells subjected to high-dose irradiation. We think that the main reason of obtaining those results is that DNA distributes in three-dimensional space. The process of image acquisition is projecting three-dimensional information onto a two-dimensional plane. DSB foci formed densely in cells exposed to high-dose radiation. Due to the limitation of the resolution of the microscope, the foci in these images connect to each other, and only a few large foci could be recognized. This effect seriously affects the accuracy of the foci counting method. For overlapping foci, the intensity of the pixels in the two-dimensional image is a physical quantity that can be superimposed. Thus, pixel intensity as a parameter can increase the accuracy of foci quantification. Many DSB repair proteins not only regulate the formation of the repair complex but also perform other biological functions in cells. Therefore, co-localization analysis was used to define the DSB response in this study, which greatly improved the accuracy of the quantification. This new method is more responsive to biological significance with regard to the recruitment of DSB response proteins to DSB sites. We found that under high-dose irradiation, the total foci area was in good agreement with the integral fluorescence intensities within this area in quantifying the foci. This result suggests that the two parameters may have some connection in the quantification of foci and can be used together to quantify DSB and repair foci formation. In future studies, different time points after irradiation or different concentrations of inhibitors could be considered to analyze the correlation between these two parameters in the quantification of DSB foci. In addition, the image analysis of cells labeled with DSB repair proteins at different time points after irradiation in combination with survival analysis could also be conducted to investigate the inhibitory effects of different specific inhibitors on DNA DSB repair.

## Conclusion

In conclusion, an alternative method was successfully established for the image-based quantification of the DNA DSB response in high-content screening studies of radiation and chemotherapy sensitizer.

## Electronic supplementary material

Supplementary Information
