# Supplementary material for: A novel automatic quantification method for high-content screening analysis of DNA double strand-break response
Source: Sci Rep. 2017 Aug 29;7:9581. doi: 10.1038/s41598-017-10063-0 (PMC5574919; doi:10.1038/s41598-017-10063-0)
Supplement: Supplementary file 1 — Supplementary Information [file 41598_2017_10063_MOESM1_ESM.pdf]

Supplementary Information

A novel automatic quantification method for high-content screening analysis of DNA double strand-break response

Jingwen Feng, Jie Lin, Pengquan Zhang, Songnan Yang, Yu Sa, Yuanming Feng

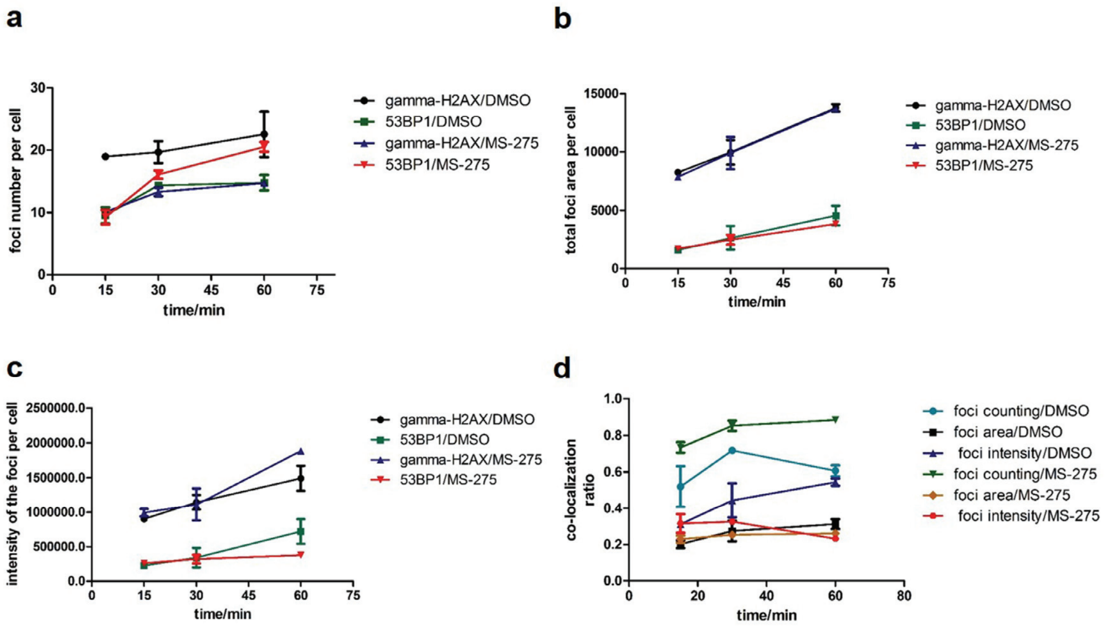

Supplementary Figure S1 Pre-experiments The materials and methods are the same as those described in the manuscript. (a) Results of foci counting method, (b) Results of foci area method, (c) Results of foci integral intensity method. (d) Co-localization ratios (53BP1 co-localized with gamma-H2AX to gamma-H2AX) calculated by those three methods. The SD values were derived from three repeated trials.

Results:

As the figures show, after 10-Gy irradiation the expression levels of gamma-H2AX and 53BP1 increased over time, and can be detected by all of these three methods (Fig.S1a, b, c).

However, the effects of MS-275 on both gamma-H2AX and 53BP1 were inconsistent with the previous reported results that MS-275 can prolong gamma-H2AX expression and inhibit 53BP1 foci formation (Fig. S1a).

Figure S1b shows that there was no obvious change in total foci area between the experimental and the control groups.

Figure S1c shows that at the time point of 60 min, 53BP1 foci formation was obviously inhibited and the expression level of gamma-H2AX increased.

The co-localization ratio is defined as the ratio of 53BP1(Co-localized with gamma-H2AX) to gamma-H2AX, which can be more sensitively detected. At the time point of 60 min, the change trends of co-localization ratio of foci area and foci intensity method were the same. But difference of the co-localization ratio between experimental and control groups calculated by foci intensity method were significant ($p<0.05$) (Fig. S1d). It was because of the inhibition of 53BP1 foci formation by MS-275 and the accumulation of gamma-H2AX expression, which is in agreement with the previous reports.[1][2] This indicates that cells cultured for 60min after irradiation can be used to compare the sensitivity of detection of the three methods on DNA DSB response.

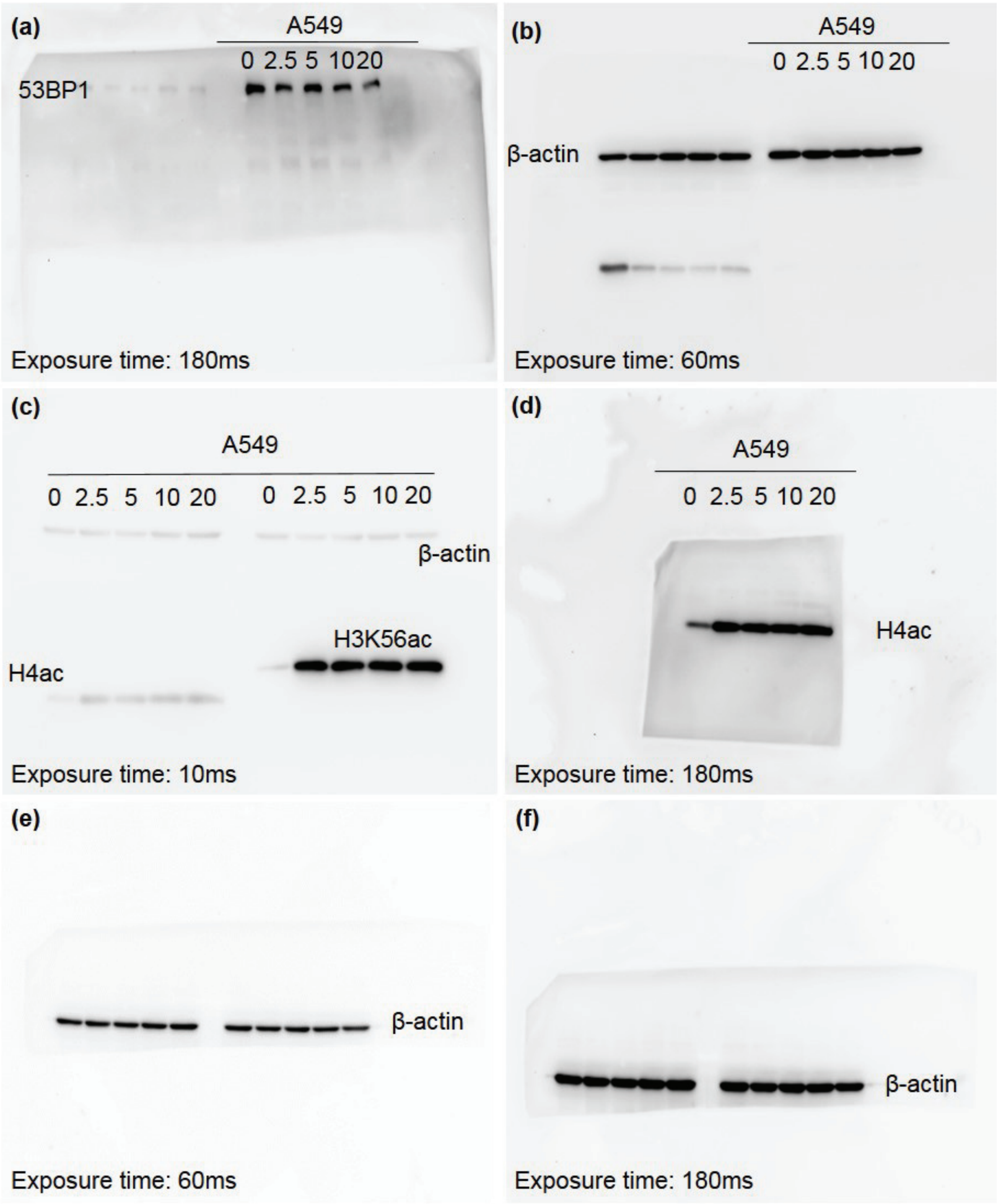

Supplementary Figure S2 Induction of H4ac and H3K56ac by MS-275 and expression level of 53BP1 in A549. (a) Expression level of 53BP1 in A549 cells treated with different concentrations of MS-275 (0 μM, 2.5 μM, 5 μM, 10 μM, 20 μM). (b) Band of β-actin corresponding to (a). (c) Expression level of H4ac and H3K56ac in A549 cells treated with different concentrations of MS-275. (d) Long exposure time image of H4ac band of (c). (e) and (f) Long exposure time image of β-actin band of (c).

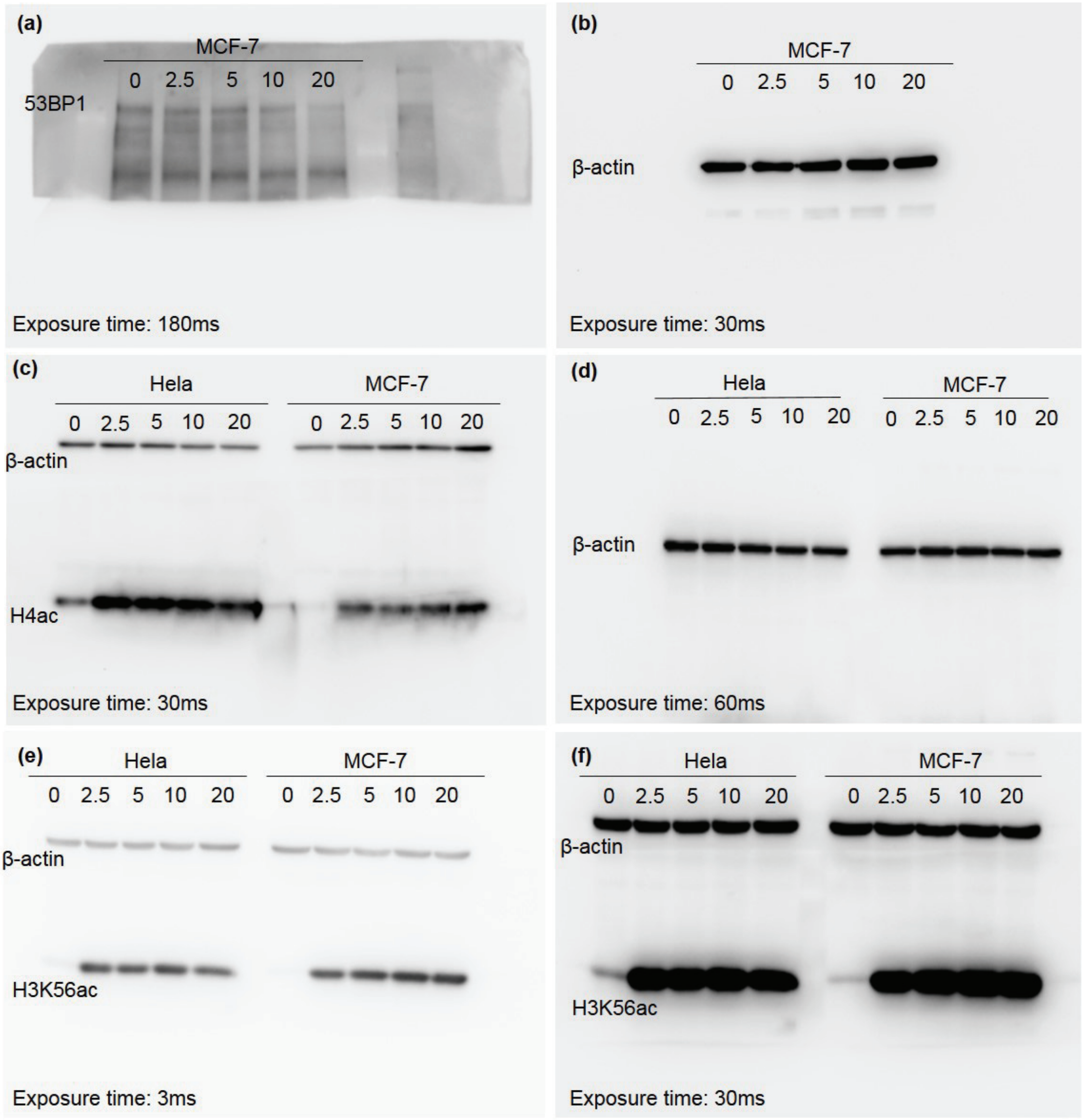

Supplementary Figure S3 Induction of H4ac and H3K56ac by MS-275 and expression level of 53BP1 in MCF-7. (a) Expression level of 53BP1 in MCF-7 cells treated with different concentrations of MS-275 (0 μM, 2.5 μM, 5 μM, 10 μM, 20 μM). (b) Band of β-actin corresponding to (a). (c) Expression level of H4ac in MCF-7 cells treated with different concentrations of MS-275. (d) Long exposure time image of β-actin band of (c). (e) Expression level of H3K56ac in MCF-7 cells treated with different concentrations of MS-275. (e) Long exposure time image of (e).
